# Comparative effects of Baduanjin and square dance on working memory, executive function performance, mood, and fatigue in older women: a randomized controlled trial

**DOI:** 10.3389/fpsyg.2026.1867615

**Published:** 2026-07-08

**Authors:** YiKun Zheng, KaiLin Mao, Lihan Lin, Guopeng Hu

**Affiliations:** 1 School of Physical Education, Huaqiao University, Quanzhou, China; 2 Provincial University Key Laboratory of Sport and Health Science, School of Physical Education and Sport Science, Fujian Normal University, Fuzhou, China

**Keywords:** Baduanjin, executive function, fatigue, healthy aging, mood, older women, randomized controlled trial, square dance

## Abstract

**Purpose:**

This exploratory randomized controlled trial compared the effects of Baduanjin and square dance on working memory, executive function performance, mood, fatigue, and selected physiological outcomes in older women.

**Methods:**

In this 12-week randomized controlled trial, 90 older women were allocated to Baduanjin, square dance, or control groups (n = 30 each). The exercise groups completed supervised 60-min sessions three times weekly. Cognitive outcomes were assessed with the N-back, Stroop, Flanker, and Digit Span tasks. Backward Digit Span and Stroop incongruent accuracy were retrospectively designated as the primary cognitive outcomes on theoretical grounds, whereas the remaining cognitive, psychological, and physiological outcomes were treated as secondary or exploratory outcomes. Seventy-nine participants completed the intervention and were included in the per-protocol analysis. Time × group interactions were tested using mixed-design ANOVA, with Bonferroni-adjusted simple-effect comparisons for interaction-significant outcomes.

**Results:**

Significant time × group interactions were observed for Stroop incongruent accuracy (*F*_(2, 76)_ = 4.916, *p* = 0.010), Flanker incongruent accuracy (*F*_(2, 76)_ = 4.683, *p* = 0.012), forward Digit Span (*F*_(2, 76)_ = 5.128, *p* = 0.008), backward Digit Span (*F*_(2, 76)_ = 6.409, *p* = 0.003), POMS score (*F*_(2, 76)_ = 3.149, *p* = 0.049), and fatigue score (*F*_(2, 76)_ = 8.212, *p* < 0.001). No significant time × group interaction was observed for any N-back outcome. In the ITT-LOCF sensitivity analysis, the main cognitive findings and fatigue result remained significant, whereas the POMS interaction was attenuated to a marginal level. Within the interaction-significant cognitive outcomes, within-group improvements were observed more consistently in the Baduanjin group, although this pattern does not establish direct superiority over square dance. For affective outcomes, both exercise groups showed favorable within-group changes. Interactions for systolic blood pressure, diastolic blood pressure, body weight, and BMI were not statistically significant.

**Conclusion:**

In this exploratory trial, Baduanjin and square dance were associated with favorable changes in selected cognitive and affective outcomes rather than across the full outcome battery. The observed cognitive pattern was descriptively broader in the Baduanjin group, but direct superiority over square dance was not established. These preliminary findings should be interpreted cautiously and require confirmation in larger, prospectively registered trials with prespecified primary outcomes.

## 1 Introduction

Population aging is accelerating worldwide, and cognitive decline has become a major challenge for healthy aging (World Health Organization, 2015, 2020). Among cognitive domains, working memory is particularly important because it supports information updating, attentional control, and goal-directed behavior in everyday life (Cansino et al., 2020). Consequently, age-related decline in working memory is closely related to reduced independence and quality of life in older adults.

Exercise has been widely recognized as a promising non-pharmacological strategy for supporting cognitive health, and regular physical activity is recommended as a core component of healthy aging (Bull et al., 2020). Previous studies have suggested that aerobic exercise, resistance training, and traditional mind-body exercise may all be associated with beneficial changes in cognitive performance among older adults (Northey et al., 2018; Bliss et al., 2023). Exercise-related cognitive benefits have also been linked to broader neuroplastic, vascular, metabolic, and inflammatory adaptations, although the relative contribution of these pathways may differ across exercise modalities (Tari et al., 2025; Lu et al., 2023; Chen et al., 2020).

Among the exercise modalities commonly practiced in Chinese communities, Baduanjin and square dance are both accessible, low-cost, and culturally acceptable. Baduanjin is a traditional Chinese mind-body exercise characterized by coordinated movement, breathing regulation, and focused attention, and it has been studied in relation to memory complaints and cognitive frailty in older adults (Su et al., 2021; Wan et al., 2022). Broader mind-body exercise literature has also reported psycho-emotional benefits in older adults (Solianik et al., 2021). By contrast, square dance is a rhythmic group-based exercise involving continuous movement, music, and social participation, and dance-based interventions have been linked to cognitive reserve, global cognition, memory, and executive function in older adults (Mitterová et al., 2021; Zhu et al., 2020; Wang et al., 2024). These distinct features suggest that the two exercise forms may not be fully interchangeable in terms of cognitive, physiological, and psychological outcomes.

Existing evidence indicates that traditional mind-body exercise may improve working memory and executive function performance in older adults (Wang et al., 2023; Wan et al., 2022; Cai et al., 2020; Wei et al., 2022). Related evidence from Tai Chi, aerobic exercise, and cardiorespiratory-fitness studies has also suggested links with working memory, inhibitory control, cerebrovascular regulation, and task-related brain responses (Håkansson et al., 2017; Guadagni et al., 2020; Hyodo et al., 2024; Cerna et al., 2024). However, most previous studies have examined Baduanjin and square dance separately, and direct comparisons between them remain limited. Comparative evidence regarding working memory and related outcomes in older women is particularly scarce. Therefore, the present study aimed to compare the effects of Baduanjin and square dance on working memory, executive function performance, mood, fatigue, and selected physiological outcomes in older women using a randomized controlled design.

## 2 Methods

### 2.1 Participants

Participants were recruited through community announcements and staff referrals at the integrated sport-health pilot workstation and the community health service center in Quanzhou, China. All participants received oral and written information about the study procedures and provided written informed consent before enrolment. The study protocol was approved by the Medical Ethics Committee of Huaqiao University Medical College (approval No. M2025004) and was conducted in accordance with the Declaration of Helsinki.

This study was not prospectively registered. The trial was designed and implemented as an investigator-initiated community-based exercise intervention under institutional ethical approval. At the time of study initiation, the research team treated the project primarily as a community exercise intervention study rather than as a prospectively registered clinical trial, which resulted in the absence of trial registration before participant enrolment. All procedures were conducted according to the approved study protocol. Nevertheless, the lack of prospective registration limits outcome-reporting transparency and should be considered when interpreting the findings, particularly because multiple cognitive, psychological, and physiological outcomes were examined.

Sample size was estimated using G^*^Power 3.1.9.7. The analysis was conducted using the *F* tests family, with the statistical test specified as ANOVA: repeated measures, within–between interaction. The calculation focused on detecting the time × group interaction. A medium effect size was assumed (Cohen's *f* = 0.25), with α = 0.05 and statistical power = 0.80. The design included three groups and two measurement time points. The assumed correlation among repeated measures was 0.50, and the non-sphericity correction was set to 1.00. The estimated minimum required sample size was 72 completers. Considering an anticipated attrition rate of approximately 20%, we planned to randomize 90 participants, with 30 participants in each group.

Inclusion criteria were as follows: female sex, age 60–70 years, ability to walk independently and complete light-to-moderate exercise, absence of known cognitive impairment or major psychiatric disorder, Montreal Cognitive Assessment (MoCA) score ≥ 26, willingness to complete the full intervention and assessment schedule, and provision of written informed consent. Exclusion criteria included known neurodegenerative disease, severe cardiovascular or musculoskeletal disease or other exercise contraindications, failure to pass the AHA/ACSM pre-exercise screening, current participation in structured exercise training, or inability to complete the required assessments or intervention procedures.

#### 2.1.1 Participant flow and baseline characteristics

A total of 90 participants were enrolled and randomly allocated to the Baduanjin group (*n* = 30), square dance group (*n* = 30), or control group (*n* = 30). During follow-up, 2 participants in the Baduanjin group, 4 in the square dance group, and 5 in the control group did not complete the study. The final per-protocol sample included 79 participants (Baduanjin: *n* = 28; square dance: *n* = 26; control: *n* = 25). Reasons for loss to follow-up or discontinuation are presented in Supplementary Table 1. No serious adverse events were reported. Attendance in both intervention groups exceeded the prespecified adherence threshold of 85%.

**Figure 1:**
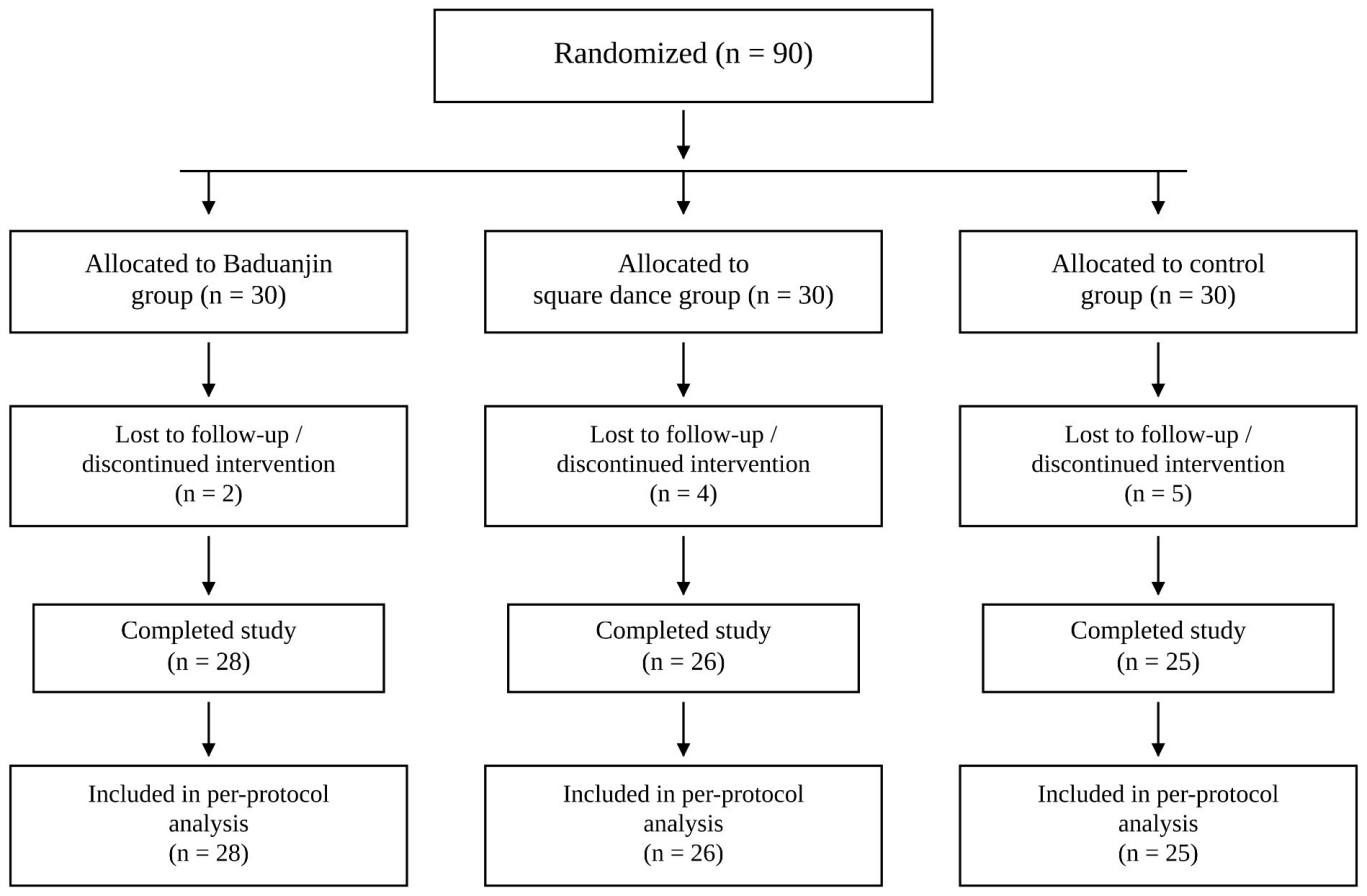
Participant flow diagram of the randomized controlled trial. Ninety participants were randomized to the Baduanjin group, square dance group, or control group (*n* = 30 each). Two participants in the Baduanjin group, four in the square dance group, and five in the control group did not complete the study. Reasons for non-completion are detailed in Supplementary Table 1. The final per-protocol sample comprised 28, 26, and 25 participants, respectively.

Figure 1 shows the participant flow through randomization, study completion, and per-protocol analysis.

Baseline demographic, physiological, and selected cognitive characteristics of the randomized sample are summarized in Table 1. No statistically significant baseline differences were observed among the three randomized groups in age, height, years of education, maximal oxygen uptake, muscle strength, heart rate, body fat percentage, or antihypertensive medication use (all *p* > 0.05), indicating acceptable baseline comparability before the intervention.

**Table 1:** Baseline demographic, physiological, and selected cognitive characteristics of the randomized participants.

Characteristic	Baduanjin group (*n* = 30)	Square dance group (*n* = 30)	Control group (*n* = 30)	*P* value
Age, years	66.0 ± 5.2	67.6 ± 5.0	67.1 ± 5.1	0.425
Height, cm	156.6 ± 4.6	154.9 ± 4.9	155.3 ± 5.3	0.367
Years of education, years	8.7 ± 2.2	8.5 ± 2.2	8.4 ± 2.1	0.863
Maximal oxygen uptake, mL/kg/min	27.1 ± 5.1	25.1 ± 4.8	25.3 ± 4.6	0.171
Muscle strength, kg	34.8 ± 7.1	32.8 ± 6.2	31.3 ± 5.8	0.094
Heart rate, beats/min	72.0 ± 8.2	74.6 ± 7.8	73.5 ± 8.1	0.331
Body fat percentage, %	28.9 ± 4.1	30.1 ± 4.0	29.5 ± 4.0	0.458
Antihypertensive medication use, n (%)	11 (36.7)	12 (40.0)	10 (33.3)	0.874
Stroop incongruent accuracy, %	92.2 ± 3.8	92.0 ± 3.9	91.8 ± 4.1	0.923
Flanker incongruent accuracy, %	90.4 ± 4.9	90.0 ± 5.0	89.9 ± 5.2	0.916
Forward digit span	5.8 ± 0.9	5.7 ± 1.0	5.9 ± 1.0	0.742
Backward digit span	4.1 ± 0.8	4.0 ± 0.9	4.2 ± 0.9	0.681

Data are presented as mean ± SD or n (%), as appropriate. *P* values refer to baseline comparisons across the three randomized groups.

Antihypertensive medication use was recorded at baseline. The proportion of participants using antihypertensive medication did not differ significantly among the three groups, and no participants reported changes in antihypertensive medication during the 12-week intervention.

Baseline comparisons between completers and non-completers are presented in Supplementary Table 2. No statistically significant baseline differences were observed between completers and non-completers, including group allocation, age, height, years of education, maximal oxygen uptake, muscle strength, heart rate, body fat percentage, or antihypertensive medication use, suggesting no clear evidence of attrition-related baseline imbalance.

### 2.2 Tests administered

All outcome assessments were conducted at baseline and after the 12-week intervention. Cognitive tests were administered individually in a quiet assessment room by trained assessors. The N-back, Stroop, and Flanker tasks were computer-based tasks programmed in E-Prime 3.0 and adapted from standard experimental paradigms, whereas the Digit Span Test was administered orally with paper-and-pencil scoring. Psychological outcomes were assessed using paper-based self-report forms. The same assessment procedures were used at baseline and post-intervention. Because no primary outcome was prospectively registered, the present analysis should be regarded as exploratory. Backward Digit Span and Stroop incongruent accuracy were retrospectively designated as the primary cognitive outcomes on theoretical grounds, representing working-memory manipulation and inhibitory control, respectively. Forward Digit Span, N-back outcomes, Flanker outcomes, POMS score, fatigue score, blood pressure, body weight, and BMI were treated as secondary or exploratory outcomes.

#### 2.2.1 N-back task

Working memory updating was assessed using computerized 1-back and 2-back tasks adapted from the standard N-back paradigm (Kirchner, 1958; Owen et al., 2005). Digits from 1 to 9 were used as visual stimuli. Each condition began with 20 practice trials to ensure that participants understood the task requirements, followed by two formal blocks of 40 trials each. Target trials accounted for approximately 30% of trials in each formal block. Each stimulus was presented for 500 ms, followed by a 1,500-ms inter-stimulus interval. Participants were instructed to respond as quickly and accurately as possible when the current digit matched the digit presented one trial earlier in the 1-back condition or two trials earlier in the 2-back condition. Reaction time for correct responses and accuracy were recorded.

#### 2.2.2 Stroop task

Inhibitory control was assessed using a computerized color-word Stroop task adapted from the classical Stroop paradigm (Stroop, 1935; MacLeod, 1991). The stimuli were Chinese color words (“红”, “黄”, “蓝”, and “绿”) presented in red, yellow, blue, or green font. In congruent trials, the semantic meaning of the color word matched the font color. In incongruent trials, the semantic meaning of the color word and the font color were inconsistent. Participants were instructed to respond according to the font color while ignoring the semantic meaning of the word. The task included 20 practice trials and 96 formal trials. Congruent and incongruent trials were presented in a mixed randomized order. Each trial began with a 500-ms fixation cross, followed by the color-word stimulus, which remained on the screen until response or for a maximum of 2,000 ms. Reaction time for correct responses and accuracy were recorded separately for congruent and incongruent conditions. No separate published Chinese validation version was used; the task was implemented as a Chinese color-word adaptation of the standard computerized Stroop paradigm.

#### 2.2.3 Flanker task

Conflict resolution and inhibitory control were assessed using a computerized arrow Flanker task adapted from the Eriksen flanker paradigm (Eriksen and Eriksen, 1974). Each stimulus array consisted of five horizontally arranged arrows, including one central target arrow and four flanking arrows, with two flankers presented on each side of the central target. In congruent trials, all arrows pointed in the same direction (e.g., “ < < < < < ” or “>>>>>”). In incongruent trials, the central target arrow pointed in the opposite direction to the flanking arrows (e.g., “ < < > < < ” or “>> < >>”). Participants were instructed to identify the direction of the central target arrow as quickly and accurately as possible while ignoring the flanking arrows. The central and flanking arrows were displayed as text stimuli in the same font and size rather than as separate image objects. Therefore, no independent physical arrow length or inter-arrow distance was programmed; adjacent arrow characters were separated by the default character spacing of the display font. The task included 20 practice trials and 96 formal trials, with congruent and incongruent trials presented in a mixed randomized order. Each trial began with a 500-ms fixation cross, followed by the arrow array, which remained on the screen until response or for a maximum of 2,000 ms. Reaction time for correct responses and accuracy were recorded separately for congruent and incongruent trials.

#### 2.2.4 Digit span test

Short-term memory and working-memory manipulation were assessed using the Digit Span Test, including forward and backward conditions, adapted from the Wechsler testing tradition and its Chinese version (Wechsler, 1997; Gong, 1992). The test was administered orally by trained assessors, and responses were recorded using paper-and-pencil scoring. In the forward condition, participants were asked to repeat digit sequences in the same order as presented. In the backward condition, participants were asked to repeat digit sequences in the reverse order. Digit sequences increased progressively in length, with two trials administered at each sequence length. Administration was discontinued when the participant failed both trials at the same sequence length. The longest correctly recalled sequence length was recorded for each condition, with higher scores indicating better performance.

#### 2.2.5 Profile of mood states

Mood was assessed using the 40-item Chinese abbreviated version of the Profile of Mood States (POMS), which was adapted from the original POMS and validated in Chinese populations (McNair et al., 1971; Zhu, 1995). The scale contains seven mood dimensions: tension, anger, fatigue, depression, vigor, confusion, and esteem-related affect. Each item is rated on a 5-point scale from 0 to 4, with higher subscale scores indicating a higher level of the corresponding mood state. The total mood disturbance score was calculated as the sum of the negative mood dimensions (tension, anger, fatigue, depression, and confusion) minus the sum of the positive mood dimensions (vigor and esteem-related affect), with a constant of 100 added for correction. A higher total mood disturbance score indicates greater negative mood disturbance.

#### 2.2.6 Fatigue score

Perceived fatigue was assessed using a single-item self-reported fatigue rating. Participants were asked to rate their perceived fatigue during the previous week on a scale from 0 to 10, where 0 indicated no fatigue and 10 indicated extreme fatigue. Higher scores represented greater perceived fatigue. This single-item rating was used as a brief field-based indicator of perceived fatigue rather than as a validated multidimensional fatigue questionnaire.

#### 2.2.7 Physiological measures

Physiological assessments included systolic blood pressure, diastolic blood pressure, body weight, body mass index (BMI), maximal oxygen uptake, muscle strength, and baseline body composition. Blood pressure was measured in a seated resting position using an Omron HEM-1026T electronic blood pressure monitor. Antihypertensive medication use was recorded at baseline, and participants were asked whether any antihypertensive medication had changed during the 12-week intervention. Body weight and height were measured using a KF-1328 device, and body composition was assessed using the InBody 120 analyzer. Maximal oxygen uptake and muscle strength were assessed according to the standardized physical-fitness testing procedures used in the study protocol. These physical-fitness variables were used for baseline characterization and were not prespecified as post-intervention outcome variables in the present analysis.

### 2.3 Procedure

Participants were randomly allocated to the Baduanjin group, the square dance group, or the control group in a 1:1:1 ratio using a computer-generated randomization sequence generated through the clinical research management platform Research Manager (ResMan). A block randomization procedure with randomly varying block sizes of 4 and 6 was used to reduce the predictability of group assignment. Group allocation was released only after completion of baseline assessment, thereby maintaining allocation concealment.

Due to the nature of the exercise interventions, participants and instructors could not be blinded to group allocation. Outcome assessors were not involved in randomization, allocation, or intervention delivery. They were instructed not to discuss group allocation with participants during assessments. However, because participants may have disclosed their exercise activities during post-intervention assessments, complete blinding of outcome assessors could not be guaranteed.

The intervention lasted 12 weeks, with supervised sessions performed three times per week for 60 min. A combined online and offline management procedure was used to monitor attendance, adherence, and health status. Offline attendance was recorded at each supervised session by research staff using a paper-based attendance sheet. Before each session, participants were asked whether they had experienced discomfort, falls, medication changes, or other health problems since the previous session. An online WeChat group was used to send session reminders, communicate schedule changes, and collect brief health-status feedback. Participants who missed a session were contacted by research staff to document the reason for absence.

Participants in the control group were asked to maintain their usual daily habits and not to participate in any new structured exercise programme during the 12-week study period. They received general health education once every 2 weeks through online messages and brief printed materials. The content covered healthy aging, balanced diet, sleep hygiene, fall prevention, chronic disease prevention, and general physical activity awareness. Each health-education contact lasted approximately 15–20 min. No supervised exercise, structured movement training, or group-based social exercise activity was provided to the control group.

The Baduanjin group practiced the standardized version promoted by the Health Qigong Management Center of the General Administration of Sport of China. Baduanjin sessions were delivered by the same instructor throughout the 12-week intervention. The instructor had experience in teaching Health Qigong and Baduanjin to older adults and received standardized training before the trial. Before the intervention, the instructor and research staff reviewed the teaching protocol to ensure consistency in session structure, movement sequence, safety reminders, and intensity control.

The square dance group performed a moderate-intensity aerobic dance routine designed for older adults, using music with a tempo of 110–130 beats/min. The choreography included simple stepping, upper-limb extension, trunk rotation, and clapping, while complex jumping and spinning movements were avoided for safety. During the main exercise phase, participants performed three to four choreographed dance routines led by the instructor through demonstration and verbal cueing, with group synchrony and social interaction encouraged throughout. Each 60-min session comprised a 10-min warm-up, 40-min main exercise phase, and 10-min cool-down. The square dance sessions were led by the same trained instructor throughout the intervention.

Exercise intensity was monitored using heart-rate spot checks. Age-predicted maximal heart rate was calculated as HRmax = 220 – age. The target intensity was 60%−70% HRmax for the Baduanjin group and 65%−75% HRmax for the square dance group. During each session, six participants in each exercise group were randomly selected for heart-rate monitoring during the main exercise phase using the Polar Verity Sense armband and Polar monitoring system. Heart rate was checked at approximately 10, 20, and 30 min of the main exercise phase. If heart rate was below or above the target range, the instructor adjusted movement amplitude, pace, or rest intervals. Heart rate was not continuously monitored in all participants throughout every session. Participants were prespecified for exclusion from the per-protocol analysis if attendance was below 85%, if they engaged in other structured exercise during the intervention period, or if they failed to complete the required assessments.

The assessment order was the same at baseline and post-intervention for all groups. Assessments were administered in a fixed order rather than counterbalanced. Participants first completed demographic and health-information collection and resting physiological measurements, followed by the computerized cognitive tasks, the orally administered Digit Span Test, the POMS questionnaire, and the fatigue rating. The same order was used in all three groups to maintain procedural consistency across assessment time points.

### 2.4 Statistical procedures

Statistical analyses were performed using SPSS 27.0. Categorical variables are presented as *n* (%) and continuous variables as mean ± standard deviation (SD). Categorical variables were analyzed using the chi-square test or Fisher's exact test, as appropriate. Continuous variables were first tested for normality and homogeneity of variance. If both assumptions were met, analysis of variance was used; otherwise, the corresponding rank-sum test was applied.

The primary analysis was conducted using the per-protocol sample. A mixed-design ANOVA was used to examine the effects of time (pre-intervention and post-intervention), group, and the time × group interaction for each outcome. In this model, time was treated as the within-subject factor and group as the between-subject factor. The time × group interaction was the primary effect of interest because it reflected whether the patterns of change over time differed among groups.

For outcomes with significant time × group interaction effects, simple-effects analyses were performed, and Bonferroni correction was applied to multiple comparisons to control the type I error rate. Cohen's *d* was used to estimate within-group effect sizes for pre-post changes. Within-group Cohen's *d* values were calculated using the mean pre-post change divided by the standard deviation of the paired differences. In the main outcome tables, the absolute values of Cohen's d are presented for outcomes with significant time × group interactions, and the direction of change is indicated by the baseline and post-intervention means. For interaction-significant outcomes, pre-to-post changes and between-group change differences were additionally summarized with 95% confidence intervals. Partial eta squared (partial η^2^) and corresponding 95% confidence intervals were reported to describe the magnitude and precision of time × group interaction effects.

To assess the robustness of the findings to attrition, an intention-to-treat sensitivity analysis was also performed using the last observation carried forward (LOCF) approach. In this analysis, all 90 randomized participants were included, and baseline values were carried forward for participants with missing post-intervention data.

All statistical analyses were conducted using a significance level of α = 0.05. Because multiple outcomes were examined, the findings—particularly those with borderline statistical significance—should be interpreted cautiously.

## 3 Results

### 3.1 Working memory and executive function outcomes

Across all assessed outcomes, statistically significant time × group interactions were observed for Stroop incongruent accuracy (*F*_(2, 76)_ = 4.916, *p* = 0.010), Flanker incongruent accuracy (*F*_(2, 76)_ = 4.683, *p* = 0.012), forward Digit Span (*F*_(2, 76)_ = 5.128, *p* = 0.008), backward Digit Span (*F*_(2, 76)_ = 6.409, *p* = 0.003), POMS score (*F*_(2, 76)_ = 3.149, *p* = 0.049), and fatigue score (*F*_(2, 76)_ = 8.212, *p* < 0.001). No significant interaction effects were observed for the remaining cognitive or physiological outcomes. Accordingly, the interpretation below focuses primarily on outcomes with statistically significant time × group interactions. For outcomes without significant interaction effects, any within-group changes are reported for descriptive completeness only and should not be interpreted as evidence of differential intervention effects.

Detailed time × group interaction results were as follows: 1-back reaction time, *F*_(2, 76)_ = 0.709, *p* = 0.496; 1-back accuracy, *F*_(2, 76)_ = 1.325, *p* = 0.272; 2-back reaction time, *F*_(2, 76)_ = 2.041, *p* = 0.137; and 2-back accuracy, *F*_(2, 76)_ = 1.179, *p* = 0.313.

For the Stroop task, the interaction results were: Stroop congruent reaction time, *F*_(2, 76)_ = 0.985, *p* = 0.378; Stroop congruent accuracy, *F*_(2, 76)_ = 2.140, *p* = 0.125; Stroop incongruent reaction time, *F*_(2, 76)_ = 1.547, *p* = 0.219; and Stroop incongruent accuracy, *F*_(2, 76)_ = 4.916, *p* = 0.010.

For the Flanker task, the interaction results were: Flanker congruent reaction time, *F*_(2, 76)_ = 0.896, *p* = 0.412; Flanker congruent accuracy, *F*_(2, 76)_ = 2.162, *p* = 0.122; Flanker incongruent reaction time, *F*_(2, 76)_ = 1.468, *p* = 0.237; and Flanker incongruent accuracy, *F*_(2, 76)_ = 4.683, *p* = 0.012. For the Digit Span Test, the interaction results were: forward Digit Span, *F*_(2, 76)_ = 5.128, *p* = 0.008; and backward Digit Span, *F*_(2, 76)_ = 6.409, *p* = 0.003.

For affective and physiological outcomes, the interaction results were: POMS score, *F*_(2, 76)_ = 3.149, *p* = 0.049; fatigue score, *F*_(2, 76)_ = 8.212, *p* < 0.001; systolic blood pressure, *F*_(2, 76)_ = 2.547, *p* = 0.085; diastolic blood pressure, *F*_(2, 76)_ = 1.868, *p* = 0.161; body weight, *F*_(2, 76)_ = 0.207, *p* = 0.814; and BMI, *F*_(2, 76)_ = 0.337, *p* = 0.715.

#### 3.1.1 N-back task

Detailed cognitive outcome data are summarized in Tables 2A, B, with full results provided in Supplementary Tables 3, 4. For all N-back indicators, cross-sectional group comparisons at baseline and post-intervention were not statistically significant (all *p* > 0.05), and no significant time × group interaction was detected. No statistically significant differential change over time was detected for any N-back outcome. Any numerically observed within-group changes should therefore be regarded as descriptive only. Thus, the N-back findings did not support differential intervention effects between the three groups in this sample.

**Table 2A:** Working memory outcomes at baseline and post-intervention in the three groups.

Outcome	Group	Baseline (M ±SD)	Post (M ±SD)	Within-group *P* value	Within-group Cohen's *d*	Group *P* at baseline	Group *P* at post
1-back reaction time (ms)	Baduanjin	700 ± 80	675 ± 75	0.032		0.814	**0.358**
	Square dance	695 ± 85	678 ± 82	0.048			
	Control	710 ± 90	705 ± 88	0.750			
1-back accuracy (%)	Baduanjin	84.5 ± 8.1	87.5 ± 7.4	0.023		0.946	**0.748**
	Square dance	85.3 ± 9.1	86.7 ± 8.3	0.051			
	Control	84.7 ± 9.2	84.9 ± 9.1	0.764			
2-back reaction time (ms)	Baduanjin	1,020 ± 115	970 ± 110	0.024		0.902	**0.305**
	Square dance	1,015 ± 120	990 ± 116	0.055			
	Control	1,030 ± 125	1,028 ± 120	0.798			
2-back accuracy (%)	Baduanjin	64.5 ± 10.8	67.5 ± 9.8	0.032		0.909	**0.379**
	Square dance	65.2 ± 11.5	68.5 ± 10.7	0.030			
	Control	63.8 ± 12.2	63.9 ± 12.0	0.876			
Forward digit span	Baduanjin	5.8 ± 0.9	6.3 ± 0.8	0.028	0.59	0.762	**0.438**
	Square dance	5.7 ± 1.0	6.0 ± 0.9	0.045	0.43		
	Control	5.9 ± 1.0	5.8 ± 1.0	0.655	0.10		
Backward digit span	Baduanjin	4.1 ± 0.8	4.6 ± 0.7	0.019	0.59	0.713	**0.356**
	Square dance	4.0 ± 0.9	4.3 ± 0.8	0.078	0.35		
	Control	4.2 ± 0.9	4.1 ± 0.9	0.721	0.13		

Data are presented as mean ± SD. Within-group *P* values indicate pre- to post-intervention comparisons and are descriptive for outcomes without significant time × group interactions. Simple-effect comparisons for interaction-significant outcomes were Bonferroni-adjusted. Group *P* values indicate cross-sectional comparisons at each time point. Cohen's d values are reported only for interaction-significant outcomes, with absolute values shown and direction indicated by the means.

**Table 2B:** Executive function outcomes at baseline and post-intervention in the three groups.

Outcome	Group	Baseline (M ±SD)	Post (M ±SD)	Within-group *P* value	Within-group Cohen's *d*	Group *P* at baseline	Group *P* at post
Stroop congruent reaction time (ms)	Baduanjin	752 ± 85	725 ± 82	0.022		0.831	0.309
	Square dance	749 ± 86	730 ± 84	0.039			
	Control	763 ± 88	760 ± 89	0.651			
Stroop congruent accuracy (%)	Baduanjin	97.8 ± 1.9	98.6 ± 1.5	0.036		0.818	0.101
	Square dance	97.6 ± 2.0	98.4 ± 1.7	0.041			
	Control	97.5 ± 2.1	97.6 ± 2.0	0.708			
Stroop incongruent reaction time (ms)	Baduanjin	952 ± 95	915 ± 93	0.010		0.862	0.296
	Square dance	949 ± 96	930 ± 94	0.083			
	Control	963 ± 98	960 ± 99	0.722			
Stroop incongruent accuracy (%)	Baduanjin	92.3 ± 3.8	94.8 ± 3.3	0.012	0.69	0.894	0.073
	Square dance	92.1 ± 3.9	93.4 ± 3.6	0.079	0.35		
	Control	91.9 ± 4.0	92.0 ± 4.0	0.761	0.03		
Flanker congruent reaction time (ms)	Baduanjin	685.5 ± 84.3	659.9 ± 85.5	0.042		0.970	0.586
	Square dance	688.4 ± 80.1	670.8 ± 83.3	0.031			
	Control	691.1 ± 83.3	688.0 ± 86.0	0.552			
Flanker congruent accuracy (%)	Baduanjin	96.8 ± 2.8	97.9 ± 2.4	0.038		0.808	0.129
	Square dance	96.5 ± 3.0	97.7 ± 2.6	0.034			
	Control	96.3 ± 3.1	96.4 ± 3.0	0.681			
Flanker incongruent reaction time (ms)	Baduanjin	885.5 ± 94.3	849.9 ± 95.5	0.045		0.976	0.490
	Square dance	888.4 ± 90.1	868.8 ± 93.3	0.035			
	Control	891.1 ± 93.3	888.0 ± 96.0	0.623			
Flanker incongruent accuracy (%)	Baduanjin	90.5 ± 4.8	93.4 ± 4.2	0.029	0.62	0.873	0.064
	Square dance	90.1 ± 5.0	92.8 ± 4.5	0.033	0.57		
	Control	89.8 ± 5.1	90.0 ± 5.0	0.602	0.05		

Data are presented as mean ± SD. Within-group *P* values refer to pre- to post-intervention comparisons within each group. For outcomes with significant time × group interactions, follow-up simple-effect comparisons were interpreted with Bonferroni adjustment. For outcomes without significant time × group interactions, within-group *P* values are presented for descriptive completeness only and should not be interpreted as evidence of intervention effectiveness or differential effects between groups. Group *P* values refer to cross-sectional comparisons across the three groups at the corresponding time point. Cohen's d values are reported only for outcomes with significant time × group interactions. Absolute values are presented, and the direction of change is indicated by the baseline and post-intervention means.

#### 3.1.2 Stroop task

For the Stroop task, cross-sectional group comparisons at baseline and post-intervention were not statistically significant (all *p* > 0.05). The time × group interaction was statistically significant for Stroop incongruent accuracy only (*F*_(2, 76)_ = 4.916, *p* = 0.010), whereas the interaction terms for Stroop congruent reaction time, Stroop congruent accuracy, and Stroop incongruent reaction time were not significant. For the only Stroop outcome with a significant time × group interaction, statistically significant within-group improvement was observed in the Baduanjin group but not in the square dance group. This result should not be interpreted as a direct between-exercise superiority finding.

#### 3.1.3 Flanker task

For the Flanker task, cross-sectional group comparisons at baseline and post-intervention were not statistically significant (all *p* > 0.05). The time × group interaction was statistically significant for Flanker incongruent accuracy (*F*_(2, 76)_ = 4.683, *p* = 0.012), but not for the remaining Flanker indicators. For the only Flanker outcome with a significant time × group interaction, statistically significant within-group improvements were observed in both exercise groups, whereas no significant change was observed in the control group. The within-group changes observed in the other Flanker outcomes should be interpreted cautiously because the corresponding interaction effects were not statistically significant.

#### 3.1.4 Digit span test

For the Digit Span Test, cross-sectional group comparisons at baseline and post-intervention were not statistically significant (all *p* > 0.05). Significant time × group interactions were observed for both forward Digit Span (*F*_(2, 76)_ = 5.128, *p* = 0.008) and backward Digit Span (*F*_(2, 76)_ = 6.409, *p* = 0.003). Among the Digit Span outcomes with significant time × group interactions, statistically significant within-group improvements were observed in both forward and backward Digit Span in the Baduanjin group, whereas in the square dance group a statistically significant within-group improvement was observed for forward Digit Span only. These patterns should not be interpreted as direct evidence of superiority of one exercise modality over the other.

Taken together, the interaction analysis indicated that differential change over time was present only for selected cognitive outcomes rather than across the full cognitive battery. Significant time × group interactions were detected for Stroop incongruent accuracy, Flanker incongruent accuracy, forward Digit Span, and backward Digit Span. Within these interaction-significant outcomes, statistically significant within-group improvements were observed more consistently in the Baduanjin group than in the square dance group. However, because the present analysis did not directly demonstrate superiority of one exercise modality over the other across these outcomes, this pattern should be interpreted as descriptive rather than definitive.

### 3.2 Psychological and physiological outcomes

Psychological and physiological outcomes are summarized in Tables 3A, B, with detailed results provided in Supplementary Tables 5, 6. Significant time × group interactions were observed for POMS score and fatigue score, whereas no significant interaction effects were detected for systolic blood pressure, diastolic blood pressure, body weight, or BMI. Changes from baseline to post-intervention and between-group change differences for outcomes with significant time × group interactions are summarized in Table 4.

**Table 3A:** Psychological outcomes at baseline and post-intervention in the three groups.

Outcome	Group	Baseline (M ±SD)	Post (M ±SD)	Within-group *P* value	Within-group Cohen's *d*	Group *P* at baseline	Group *P* at post
POMS score	Baduanjin	45.2 ± 8.4	40.8 ± 8.5	0.037	0.53	0.590	**0.719**
	Square dance	44.1 ± 8.9	39.2 ± 7.8	0.011	0.57		
	Control	42.7 ± 9.2	41.9 ± 9.0	0.428	0.16		
Fatigue score	Baduanjin	6.5 ± 1.8	6.1 ± 1.7	0.015	0.52	0.639	**0.686**
	Square dance	6.8 ± 1.9	5.2 ± 1.6	< 0.001	1.18		
	Control	6.3 ± 2.0	6.1 ± 1.9	0.382	0.17		

Data are presented as mean ± SD. Within-group *P* values refer to pre- to post-intervention comparisons within each group. Within-group Cohen's *d* values were calculated using the mean pre-post change divided by the standard deviation of the paired differences, and absolute values are presented. Group *P* values refer to cross-sectional comparisons across the three groups at the corresponding time point. For outcomes with significant time × group interactions, follow-up simple-effect comparisons were interpreted with Bonferroni adjustment.

**Table 3B:** Physiological outcomes at baseline and post-intervention in the three groups.

Outcome	Group	Baseline (M ±SD)	Post (M ±SD)	Within-group *P* value	Group *P* at baseline	Group *P* at post
Systolic blood pressure (mmHg)	Baduanjin	143.5 ± 7.2	140.5 ± 6.9	0.032	0.725	0.699
	Square dance	142.8 ± 7.5	138.9 ± 7.1	0.008		
	Control	141.9 ± 7.0	141.2 ± 6.8	0.401		
Diastolic blood pressure (mmHg)	Baduanjin	88.5 ± 5.8	86.5 ± 5.5	0.041	0.752	0.682
	Square dance	87.9 ± 5.6	85.2 ± 5.3	0.012		
	Control	87.3 ± 5.9	86.8 ± 5.7	0.485		
Body weight (kg)	Baduanjin	58.2 ± 6.8	57.5 ± 6.6	0.065	0.896	0.929
	Square dance	58.8 ± 7.2	57.8 ± 7.0	0.018		
	Control	57.9 ± 7.0	57.8 ± 6.9	0.725		
BMI (kg/m^2^)	Baduanjin	23.9 ± 2.3	23.6 ± 2.2	0.062	0.824	0.883
	Square dance	24.2 ± 2.5	23.7 ± 2.4	0.014		
	Control	23.8 ± 2.4	23.7 ± 2.4	0.695		

Data are presented as mean ± SD. Within-group *P* values refer to pre- to post-intervention comparisons within each group. For outcomes with significant time × group interactions, follow-up simple-effect comparisons were interpreted with Bonferroni adjustment. For outcomes without significant time × group interactions, within-group *P* values are presented for descriptive completeness only and should not be interpreted as evidence of intervention effectiveness or differential effects between groups. Group *P* values refer to cross-sectional comparisons across the three groups at the corresponding time point.

**Table 4:** Changes and between-group change differences for interaction-significant outcomes.

Outcome	Baduanjin change 95% CI	Square dance change 95% CI	Control change 95% CI	Baduanjin vs. control difference 95% CI	Square dance vs. control difference 95% CI	Baduanjin vs. Square dance difference 95% CI
Stroop incongruent accuracy, %	+2.5 [1.1, 3.9]	+1.3 [−0.2, 2.8]	+0.1 [−1.3, 1.5]	+2.4 [0.5, 4.3]	+1.2 [−0.8, 3.2]	+1.2 [−0.8, 3.2]
Flanker incongruent accuracy, %	+2.9 [1.1, 4.7]	+2.7 [0.8, 4.6]	+0.2 [−1.5, 1.9]	+2.7 [0.3, 5.1]	+2.5 [0.1, 4.9]	+0.2 [−2.3, 2.7]
Forward digit span	+0.52 [0.18, 0.86]	+0.33 [0.02, 0.64]	−0.07 [−0.36, 0.22]	+0.59 [0.15, 1.03]	+0.40 [−0.02, 0.82]	+0.19 [−0.25, 0.63]
Backward digit span	+0.49 [0.17, 0.81]	+0.28 [−0.04, 0.60]	−0.09 [−0.38, 0.20]	+0.58 [0.15, 1.01]	+0.37 [−0.06, 0.80]	+0.21 [−0.24, 0.66]
POMS score	−4.4 [−7.6, −1.2]	−4.9 [−8.4, −1.4]	−0.8 [−2.9, 1.3]	−3.6 [−7.2, 0.0]	−4.1 [−7.9, −0.3]	+0.5 [−4.3, 5.3]
Fatigue score	−0.4 [−0.7, −0.1]	−1.6 [−2.2, −1.1]	−0.2 [−0.7, 0.3]	−0.2 [−0.8, 0.4]	−1.4 [−2.1, −0.7]	+1.2 [0.5, 1.9]

Values are mean changes from baseline to post-intervention with 95% confidence intervals. Positive values indicate increases, whereas negative values indicate decreases. Between-group differences were calculated as differences in mean change scores. For POMS and fatigue scores, negative changes indicate improvement.

For psychological outcomes, the significant time × group interactions for POMS and fatigue indicate that change over time differed across groups for these two outcomes, and both exercise groups showed favorable within-group changes. In the Baduanjin group, POMS scores decreased from 45.2 ± 8.4 to 40.8 ± 8.5 (*p* = 0.037), and fatigue scores decreased from 6.5 ± 1.8 to 6.1 ± 1.7 (*p* = 0.015). In the square dance group, POMS scores decreased from 44.1 ± 8.9 to 39.2 ± 7.8 (*p* = 0.011), and fatigue scores decreased from 6.8 ± 1.9 to 5.2 ± 1.6 (*p* < 0.001). No significant changes were observed in the control group.

For physiological outcomes, within-group improvements were observed in some comparisons, particularly for blood pressure in both exercise groups and for body weight and BMI in the square dance group. In the Baduanjin group, systolic blood pressure decreased from 143.5 ± 7.2 mmHg to 140.5 ± 6.9 mmHg (*p* = 0.032), and diastolic blood pressure decreased from 88.5 ± 5.8 mmHg to 86.5 ± 5.5 mmHg (*p* = 0.041). In the square dance group, systolic blood pressure decreased from 142.8 ± 7.5 mmHg to 138.9 ± 7.1 mmHg (*p* = 0.008), and diastolic blood pressure decreased from 87.9 ± 5.6 mmHg to 85.2 ± 5.3 mmHg (*p* = 0.012). The square dance group also showed significant within-group reductions in body weight and BMI. However, because the time × group interactions for these physiological outcomes were not statistically significant, these within-group changes should be interpreted as descriptive within-group findings rather than as evidence of clear differential effects between interventions.

### 3.3 Sensitivity analysis

An intention-to-treat sensitivity analysis using LOCF was conducted in all 90 randomized participants. The significant time × group interactions for Stroop incongruent accuracy, Flanker incongruent accuracy, forward Digit Span, backward Digit Span, and fatigue score remained statistically significant. The interaction for POMS score was attenuated to a marginal level in the ITT-LOCF analysis (*F*_(2, 76)_ = 2.73, *p* = 0.071). Interactions for systolic blood pressure, diastolic blood pressure, body weight, and BMI remained non-significant. These findings suggest that the main cognitive findings and fatigue result were relatively robust to attrition, whereas the POMS finding should be interpreted with greater caution.

## 4 Discussion

### 4.1 Principal findings

The present study compared the effects of Baduanjin and square dance on working memory, executive function performance, mood, fatigue, and selected physiological outcomes in older women. Two main findings emerged. First, statistically significant time × group interactions were observed for only a limited subset of outcomes, namely Stroop incongruent accuracy, Flanker incongruent accuracy, forward Digit Span, backward Digit Span, POMS score, and fatigue score, whereas the remaining outcomes did not show significant interaction effects. Second, within the interaction-significant cognitive outcomes, statistically significant within-group improvements were observed more consistently in the Baduanjin group than in the square dance group, whereas both exercise groups demonstrated favorable changes in perceived fatigue. However, the POMS finding was less robust because the interaction was attenuated to a marginal level in the ITT-LOCF sensitivity analysis. The present results also do not support clear differential effects on blood pressure, body weight, or BMI because the interaction terms for these outcomes were not statistically significant.

These findings suggest that exercise-related responses in older women may differ across specific cognitive and affective domains rather than uniformly across all measured outcomes. In practical terms, both community-based exercise modalities appeared promising, but the observed patterns should be interpreted cautiously and confirmed in larger studies.

### 4.2 Interpretation of cognitive findings

As indicated by the interaction analysis, significant group differences in change over time were observed only for selected cognitive outcomes rather than across the full cognitive battery. Among these interaction-significant measures, statistically significant within-group improvements across the interaction-significant cognitive outcomes were observed more consistently in the Baduanjin group than in the square dance group. In the present sample, this pattern should not be interpreted as conclusive evidence that Baduanjin was superior to square dance.

A cautious interpretation is that the coordinated movement sequences, breathing regulation, and attentional engagement involved in Baduanjin practice may be relevant to the pattern observed in selected cognitive outcomes. However, the present study did not include mechanistic measures, and such explanations should be regarded only as background interpretation rather than direct evidence.

### 4.3 Comparison with previous studies

The present findings are broadly consistent with, but also more circumscribed than, previous evidence on exercise-related cognitive and affective outcomes in older adults. The more consistent within-group improvements in selected interaction-significant cognitive outcomes in the Baduanjin group align with earlier studies suggesting that traditional mind-body exercise may support working memory and executive function performance in older adults (Cai et al., 2020; Wang et al., 2023; Wan et al., 2022; Wei et al., 2022). These effects may plausibly relate to the combined attentional, breathing, postural-control, and motor-coordination demands of Baduanjin. However, because the present trial did not demonstrate consistent direct superiority of Baduanjin over square dance, this pattern should be interpreted as descriptive rather than definitive.

The square dance results should be interpreted in relation to the broader dance-based exercise literature. Wang et al. (2024) reported that a 12-week square dance intervention improved global cognitive function and executive-function-related domains in older adults with mild cognitive impairment, with balance ability and executive function partly mediating the cognitive benefits. Similarly, Zhu et al. (2020) found in a systematic review and meta-analysis that aerobic dance improved global cognition, memory, and executive function in older adults with mild cognitive impairment. Together with evidence linking dance interventions to cognitive reserve and healthy aging in older populations (Mitterová et al., 2021), these studies support the plausibility that rhythmic, music-accompanied, and socially embedded dance exercise may contribute to cognitive benefits through combined aerobic, coordinative, and social-cognitive stimulation. Because the present participants were community-dwelling older women without diagnosed cognitive impairment, these comparisons should be regarded as supportive rather than directly equivalent evidence.

The affective findings are also broadly compatible with previous work suggesting that rhythmic, socially engaging, and music-accompanied exercise may be associated with favorable psychological outcomes in older adults. In the present study, both exercise groups showed favorable within-group changes in affective outcomes, while fatigue reduction was numerically larger in the square dance group than in the Baduanjin group. This pattern may reflect the rhythmic, aerobic, and socially interactive characteristics of square dance. However, because exercise enjoyment, social connectedness, sleep quality, and daily activity outside the intervention were not directly measured, this explanation remains speculative. Therefore, the affective and fatigue-related findings should be interpreted cautiously and confirmed in future trials that include more detailed psychosocial and behavioral measures.

### 4.4 Interpretation of physiological and psychological findings

For psychological outcomes, the significant time × group interactions for POMS and fatigue in the per-protocol analysis indicate that change over time differed across groups for these two outcomes. However, the affective findings should be interpreted with different levels of caution. The fatigue result remained statistically significant in the ITT-LOCF sensitivity analysis, whereas the POMS interaction was attenuated to a marginal level. These results suggest that both Baduanjin and square dance may be associated with favorable affective changes, but the robustness of the POMS finding is limited.

It should also be noted that fatigue reduction differed in magnitude between the two exercise groups. The square dance group showed a larger numerical decrease in perceived fatigue than the Baduanjin group, which may reflect the more aerobic, rhythmic, and socially engaging characteristics of square dance. However, because exercise enjoyment, social interaction, sleep quality, and daily activity outside the intervention were not directly measured, this explanation remains speculative. In addition, perceived fatigue was assessed using a single-item rating rather than a validated multidimensional fatigue questionnaire, which limits clinical interpretation of the fatigue result.

By contrast, for physiological outcomes, several within-group improvements were observed, particularly in blood pressure in both exercise groups and in body weight and BMI in the square dance group. However, the interaction effects for systolic blood pressure, diastolic blood pressure, body weight, and BMI were not statistically significant. Accordingly, these findings should be interpreted as within-group changes rather than as evidence of clear between-group differences in physiological response patterns. Although antihypertensive medication use was balanced across groups at baseline and no medication changes were reported during the intervention, blood pressure findings should still be interpreted cautiously because medication type, dosage, adherence, and broader lifestyle factors were not analyzed in detail.

### 4.5 Practical implications

Both Baduanjin and square dance are accessible, low-cost, and feasible forms of community-based exercise for older women. The present findings support their potential value as community health-promotion activities, particularly with regard to selected cognitive and affective outcomes. Within the cognitive outcomes that showed significant time × group interactions, statistically significant within-group improvements were observed more consistently in the Baduanjin group; however, this pattern is not sufficient to justify a firm recommendation of one exercise modality over the other. The present results also do not support strong claims regarding differential effects on blood pressure, body weight, or BMI.

### 4.6 Limitations and future directions

Several limitations should be acknowledged. First, the sample size was modest, which may have reduced statistical power and the precision of the estimates. Second, only older women were included, and therefore the findings may not be generalizable to men or to more clinically diverse populations. Third, the intervention period was limited to 12 weeks, and no long-term follow-up was performed. Fourth, the study was not prospectively registered, and no primary outcome was prespecified before enrolment; therefore, the outcome hierarchy used in the revised manuscript was designated retrospectively and the findings should be regarded as exploratory. Fifth, multiple outcomes were tested, and some findings, particularly those close to the significance threshold, may be susceptible to type I error inflation. Sixth, although an ITT-LOCF sensitivity analysis was performed, LOCF is a simple imputation approach and may not fully address attrition-related bias. Seventh, the POMS finding was not robust in the ITT-LOCF sensitivity analysis and should therefore be interpreted cautiously. Eighth, perceived fatigue was assessed using a single-item rating rather than a validated multidimensional fatigue questionnaire; therefore, clinical interpretation of the fatigue change should be cautious. Ninth, the assessment order was fixed rather than counterbalanced, and therefore potential practice or fatigue effects across tasks could not be fully controlled. Tenth, the study did not include neural, vascular, biomarker-based, enjoyment, social-interaction, or sleep-related measures, which limits mechanistic interpretation. Finally, although antihypertensive medication use and medication changes were recorded, medication type, dosage, adherence, and broader lifestyle factors were not analyzed in detail, which limits interpretation of blood pressure outcomes.

Future studies should include larger and more diverse samples, longer intervention periods, and long-term follow-up assessments. Future trials should also be prospectively registered, prespecify primary outcomes, include more rigorous intention-to-treat approaches such as multiple imputation where appropriate, and use validated multidimensional measures for fatigue and psychological outcomes. It would also be valuable to investigate the neural, vascular, behavioral, and social mechanisms underlying different exercise modalities. Such work would help determine whether the differential patterns observed here are reproducible and provide stronger theoretical and practical guidance for community-based exercise interventions in older adults.

## 5 Conclusion

This exploratory randomized controlled trial suggests that Baduanjin and square dance may be associated with favorable changes in selected cognitive and affective outcomes among older women, rather than across the full outcome battery. Significant time × group interactions were observed for Stroop incongruent accuracy, Flanker incongruent accuracy, forward Digit Span, backward Digit Span, POMS score, and fatigue score in the per-protocol analysis, whereas no significant interaction was observed for any N-back outcome. The main cognitive findings and fatigue result were generally supported by the ITT-LOCF sensitivity analysis, whereas the POMS finding was attenuated to a marginal level and should therefore be interpreted with caution. Within the interaction-significant cognitive outcomes, within-group improvements were observed more consistently in the Baduanjin group than in the square dance group; however, this pattern should not be interpreted as direct evidence of superiority. Physiological outcomes showed some descriptive within-group changes, but the corresponding time × group interactions were not statistically significant. These findings should be interpreted cautiously and require confirmation in larger, prospectively registered trials with prespecified primary outcomes and more rigorous intention-to-treat analyses.

## Data Availability

The raw data supporting the conclusions of this article will be made available by the authors, without undue reservation.
