# Supplementary material for: Comparative effects of Baduanjin and square dance on working memory, executive function performance, mood, and fatigue in older women: a randomized controlled trial
Source: Front Psychol. 2026 Jul 8;17:1867615. doi: 10.3389/fpsyg.2026.1867615 (PMC13390624; doi:10.3389/fpsyg.2026.1867615)
Supplement: Supplementary file 1 [file Supplementary_file_1.docx]

**Supplementary Material**

**Supplementary Table S1. Reasons for loss to follow-up or discontinuation**

| **Group** | **Randomized** | **Did not complete** | **Reasons** |
| --- | --- | --- | --- |
| Baduanjin | 30 | 2 | Time conflict/family caregiving (n = 1); relocation (n = 1) |
| Square dance | 30 | 4 | Time conflict (n = 2); personal reason (n = 1); mild knee discomfort unrelated to intervention (n = 1) |
| Control | 30 | 5 | Lost to follow-up (n = 2); time conflict (n = 2); family reason (n = 1) |
| Total | 90 | 11 | No serious adverse events were reported. |

**Note.** Reasons are reported as recorded by the research staff during follow-up. The reported mild knee discomfort in the square dance group was judged to be unrelated to the intervention. No serious adverse events were reported.

**Supplementary Table S2. Baseline comparison between completers and non-completers**

| **Variable** | **Completers (n = 79)** | **Non-completers (n = 11)** | **P value** |
| --- | --- | --- | --- |
| Group allocation, Baduanjin/Square dance/Control | 28/26/25 | 2/4/5 | 0.551 |
| Age, years | 66.9 ± 5.2 | 66.6 ± 5.5 | 0.833 |
| Height, cm | 155.7 ± 4.9 | 155.8 ± 5.1 | 0.951 |
| Years of education, years | 8.6 ± 2.2 | 8.5 ± 2.4 | 0.918 |
| Maximal oxygen uptake, mL/kg/min | 25.9 ± 5.0 | 25.7 ± 4.9 | 0.932 |
| Muscle strength, kg | 33.0 ± 6.5 | 32.6 ± 6.2 | 0.846 |
| Heart rate, beats/min | 73.4 ± 8.1 | 73.1 ± 8.5 | 0.910 |
| Body fat percentage, % | 29.5 ± 4.1 | 29.8 ± 4.2 | 0.811 |
| Antihypertensive medication use, n (%) | 30 (38.0) | 3 (27.3) | 0.487 |

**Note.** Data are presented as mean ± SD, n (%), or counts, as appropriate. P values refer to comparisons between completers and non-completers. Antihypertensive medication use was recorded at baseline; no participants reported changes in antihypertensive medication during the 12-week intervention.

**Supplementary Table S3. Detailed within-group pre-post comparisons of cognitive outcomes**

| **Outcome** | **Group** | **Pre-intervention (M ± SD)** | **Post-intervention (M ± SD)** | **Within-group P value** |
| --- | --- | --- | --- | --- |
| 1-back reaction time (ms) | Baduanjin | 700 ± 80 | 675 ± 75 | 0.032 |
|  | Square dance | 695 ± 85 | 678 ± 82 | 0.048 |
|  | Control | 710 ± 90 | 705 ± 88 | 0.750 |
| 1-back accuracy (%) | Baduanjin | 84.5 ± 8.1 | 87.5 ± 7.4 | 0.023 |
|  | Square dance | 85.3 ± 9.1 | 86.7 ± 8.3 | 0.051 |
|  | Control | 84.7 ± 9.2 | 84.9 ± 9.1 | 0.764 |
| 2-back reaction time (ms) | Baduanjin | 1020 ± 115 | 970 ± 110 | 0.024 |
|  | Square dance | 1015 ± 120 | 990 ± 116 | 0.055 |
|  | Control | 1030 ± 125 | 1028 ± 120 | 0.798 |
| 2-back accuracy (%) | Baduanjin | 64.5 ± 10.8 | 67.5 ± 9.8 | 0.032 |
|  | Square dance | 65.2 ± 11.5 | 68.5 ± 10.7 | 0.030 |
|  | Control | 63.8 ± 12.2 | 63.9 ± 12.0 | 0.876 |
| Stroop congruent reaction time (ms) | Baduanjin | 752 ± 85 | 725 ± 82 | 0.022 |
|  | Square dance | 749 ± 86 | 730 ± 84 | 0.039 |
|  | Control | 763 ± 88 | 760 ± 89 | 0.651 |
| Stroop congruent accuracy (%) | Baduanjin | 97.8 ± 1.9 | 98.6 ± 1.5 | 0.036 |
|  | Square dance | 97.6 ± 2.0 | 98.4 ± 1.7 | 0.041 |
|  | Control | 97.5 ± 2.1 | 97.6 ± 2.0 | 0.708 |
| Stroop incongruent reaction time (ms) | Baduanjin | 952 ± 95 | 915 ± 93 | 0.010 |
|  | Square dance | 949 ± 96 | 930 ± 94 | 0.083 |
|  | Control | 963 ± 98 | 960 ± 99 | 0.722 |
| Stroop incongruent accuracy (%) | Baduanjin | 92.3 ± 3.8 | 94.8 ± 3.3 | 0.012 |
|  | Square dance | 92.1 ± 3.9 | 93.4 ± 3.6 | 0.079 |
|  | Control | 91.9 ± 4.0 | 92.0 ± 4.0 | 0.761 |

**Supplementary Table S3 (continued).**

| **Outcome** | **Group** | **Pre-intervention (M ± SD)** | **Post-intervention (M ± SD)** | **Within-group P value** |
| --- | --- | --- | --- | --- |
| Flanker congruent reaction time (ms) | Baduanjin | 685.5 ± 84.3 | 659.9 ± 85.5 | 0.042 |
|  | Square dance | 688.4 ± 80.1 | 670.8 ± 83.3 | 0.031 |
|  | Control | 691.1 ± 83.3 | 688.0 ± 86.0 | 0.552 |
| Flanker congruent accuracy (%) | Baduanjin | 96.8 ± 2.8 | 97.9 ± 2.4 | 0.038 |
|  | Square dance | 96.5 ± 3.0 | 97.7 ± 2.6 | 0.034 |
|  | Control | 96.3 ± 3.1 | 96.4 ± 3.0 | 0.681 |
| Flanker incongruent reaction time (ms) | Baduanjin | 885.5 ± 94.3 | 849.9 ± 95.5 | 0.045 |
|  | Square dance | 888.4 ± 90.1 | 868.8 ± 93.3 | 0.035 |
|  | Control | 891.1 ± 93.3 | 888.0 ± 96.0 | 0.623 |
| Flanker incongruent accuracy (%) | Baduanjin | 90.5 ± 4.8 | 93.4 ± 4.2 | 0.029 |
|  | Square dance | 90.1 ± 5.0 | 92.8 ± 4.5 | 0.033 |
|  | Control | 89.8 ± 5.1 | 90.0 ± 5.0 | 0.602 |
| Forward Digit Span | Baduanjin | 5.8 ± 0.9 | 6.3 ± 0.8 | 0.028 |
|  | Square dance | 5.7 ± 1.0 | 6.0 ± 0.9 | 0.045 |
|  | Control | 5.9 ± 1.0 | 5.8 ± 1.0 | 0.655 |
| Backward Digit Span | Baduanjin | 4.1 ± 0.8 | 4.6 ± 0.7 | 0.019 |
|  | Square dance | 4.0 ± 0.9 | 4.3 ± 0.8 | 0.078 |
|  | Control | 4.2 ± 0.9 | 4.1 ± 0.9 | 0.721 |

**Note.** Data are presented as mean ± SD. Within-group P values refer to pre- to post-intervention comparisons within each group. For outcomes with significant time × group interactions, follow-up simple-effect comparisons were interpreted with Bonferroni adjustment. For outcomes without significant time × group interactions, within-group P values are shown for descriptive completeness only and should not be interpreted as evidence of differential intervention effects.

**Supplementary Table S4. Cross-sectional group comparisons of cognitive outcomes at baseline and post-intervention**

| **Outcome** | **Baseline Baduanjin (M ± SD)** | **Baseline Square dance (M ± SD)** | **Baseline Control (M ± SD)** | **F baseline** | **P baseline** | **Post-intervention Baduanjin (M ± SD)** | **Post-intervention Square dance (M ± SD)** | **Post-intervention Control (M ± SD)** | **F post-intervention** | **P post-intervention** |
| --- | --- | --- | --- | --- | --- | --- | --- | --- | --- | --- |
| 1-back reaction time (ms) | 700 ± 80 | 695 ± 85 | 710 ± 90 | 0.206 | 0.814 | 675 ± 75 | 678 ± 82 | 705 ± 88 | 1.036 | 0.358 |
| 1-back accuracy (%) | 84.5 ± 8.1 | 85.3 ± 9.1 | 84.7 ± 9.2 | 0.055 | 0.946 | 87.5 ± 7.4 | 86.7 ± 8.3 | 84.9 ± 9.1 | 0.291 | 0.748 |
| 2-back reaction time (ms) | 1020 ± 115 | 1015 ± 120 | 1030 ± 125 | 0.103 | 0.902 | 970 ± 110 | 990 ± 116 | 1028 ± 120 | 1.197 | 0.305 |
| 2-back accuracy (%) | 64.5 ± 10.8 | 65.2 ± 11.5 | 63.8 ± 12.2 | 0.095 | 0.909 | 67.5 ± 9.8 | 68.5 ± 10.7 | 63.9 ± 12.0 | 0.976 | 0.379 |
| Stroop congruent reaction time (ms) | 752 ± 85 | 749 ± 86 | 763 ± 88 | 0.186 | 0.831 | 725 ± 82 | 730 ± 84 | 760 ± 89 | 1.184 | 0.309 |
| Stroop congruent accuracy (%) | 97.8 ± 1.9 | 97.6 ± 2.0 | 97.5 ± 2.1 | 0.201 | 0.818 | 98.6 ± 1.5 | 98.4 ± 1.7 | 97.6 ± 2.0 | 2.368 | 0.101 |
| Stroop incongruent reaction time (ms) | 952 ± 95 | 949 ± 96 | 963 ± 98 | 0.149 | 0.862 | 915 ± 93 | 930 ± 94 | 960 ± 99 | 1.228 | 0.296 |
| Stroop incongruent accuracy (%) | 92.3 ± 3.8 | 92.1 ± 3.9 | 91.9 ± 4.0 | 0.112 | 0.894 | 94.8 ± 3.3 | 93.4 ± 3.6 | 92.0 ± 4.0 | 2.714 | 0.073 |
| Flanker congruent reaction time (ms) | 685.5 ± 84.3 | 688.4 ± 80.1 | 691.1 ± 83.3 | 0.030 | 0.970 | 659.9 ± 85.5 | 670.8 ± 83.3 | 688.0 ± 86.0 | 0.537 | 0.586 |
| Flanker congruent accuracy (%) | 96.8 ± 2.8 | 96.5 ± 3.0 | 96.3 ± 3.1 | 0.214 | 0.808 | 97.9 ± 2.4 | 97.7 ± 2.6 | 96.4 ± 3.0 | 2.105 | 0.129 |
| Flanker incongruent reaction time (ms) | 885.5 ± 94.3 | 888.4 ± 90.1 | 891.1 ± 93.3 | 0.024 | 0.976 | 849.9 ± 95.5 | 868.8 ± 93.3 | 888.0 ± 96.0 | 0.718 | 0.490 |
| Flanker incongruent accuracy (%) | 90.5 ± 4.8 | 90.1 ± 5.0 | 89.8 ± 5.1 | 0.136 | 0.873 | 93.4 ± 4.2 | 92.8 ± 4.5 | 90.0 ± 5.0 | 2.864 | 0.064 |
| Forward Digit Span | 5.8 ± 0.9 | 5.7 ± 1.0 | 5.9 ± 1.0 | 0.273 | 0.762 | 6.3 ± 0.8 | 6.0 ± 0.9 | 5.8 ± 1.0 | 0.830 | 0.438 |
| Backward Digit Span | 4.1 ± 0.8 | 4.0 ± 0.9 | 4.2 ± 0.9 | 0.340 | 0.713 | 4.6 ± 0.7 | 4.3 ± 0.8 | 4.1 ± 0.9 | 1.040 | 0.356 |

**Note.** Data are presented as mean ± SD. F and P values refer to cross-sectional group comparisons across the three groups at the corresponding time point and do not represent the time × group interaction from the mixed-design ANOVA.

**Supplementary Table S5. Detailed within-group pre-post comparisons of psychological and physiological outcomes**

| **Outcome** | **Group** | **Pre-intervention (M ± SD)** | **Post-intervention (M ± SD)** | **Within-group P value** |
| --- | --- | --- | --- | --- |
| POMS score | Baduanjin | 45.2 ± 8.4 | 40.8 ± 8.5 | 0.037 |
|  | Square dance | 44.1 ± 8.9 | 39.2 ± 7.8 | 0.011 |
|  | Control | 42.7 ± 9.2 | 41.9 ± 9.0 | 0.428 |
| Fatigue score | Baduanjin | 6.5 ± 1.8 | 6.1 ± 1.7 | 0.015 |
|  | Square dance | 6.8 ± 1.9 | 5.2 ± 1.6 | <0.001 |
|  | Control | 6.3 ± 2.0 | 6.1 ± 1.9 | 0.382 |
| Systolic blood pressure (mmHg) | Baduanjin | 143.5 ± 7.2 | 140.5 ± 6.9 | 0.032 |
|  | Square dance | 142.8 ± 7.5 | 138.9 ± 7.1 | 0.008 |
|  | Control | 141.9 ± 7.0 | 141.2 ± 6.8 | 0.401 |
| Diastolic blood pressure (mmHg) | Baduanjin | 88.5 ± 5.8 | 86.5 ± 5.5 | 0.041 |
|  | Square dance | 87.9 ± 5.6 | 85.2 ± 5.3 | 0.012 |
|  | Control | 87.3 ± 5.9 | 86.8 ± 5.7 | 0.485 |
| Body weight (kg) | Baduanjin | 58.2 ± 6.8 | 57.5 ± 6.6 | 0.065 |
|  | Square dance | 58.8 ± 7.2 | 57.8 ± 7.0 | 0.018 |
|  | Control | 57.9 ± 7.0 | 57.8 ± 6.9 | 0.725 |
| BMI (kg/m²) | Baduanjin | 23.9 ± 2.3 | 23.6 ± 2.2 | 0.062 |
|  | Square dance | 24.2 ± 2.5 | 23.7 ± 2.4 | 0.014 |
|  | Control | 23.8 ± 2.4 | 23.7 ± 2.4 | 0.695 |

**Note.** Data are presented as mean ± SD. Within-group P values refer to pre- to post-intervention comparisons within each group. For outcomes with significant time × group interactions, follow-up simple-effect comparisons were interpreted with Bonferroni adjustment. For outcomes without significant time × group interactions, within-group P values are shown for descriptive completeness only and should not be interpreted as evidence of differential intervention effects.

**Supplementary Table S6. Cross-sectional group comparisons of psychological and physiological outcomes at baseline and post-intervention**

| **Outcome** | **Baseline Baduanjin (M ± SD)** | **Baseline Square dance (M ± SD)** | **Baseline Control (M ± SD)** | **F baseline** | **P baseline** | **Post-intervention Baduanjin (M ± SD)** | **Post-intervention Square dance (M ± SD)** | **Post-intervention Control (M ± SD)** | **F post-intervention** | **P post-intervention** |
| --- | --- | --- | --- | --- | --- | --- | --- | --- | --- | --- |
| POMS score | 45.2 ± 8.4 | 44.1 ± 8.9 | 42.7 ± 9.2 | 0.530 | 0.590 | 40.8 ± 8.5 | 39.2 ± 7.8 | 41.9 ± 9.0 | 0.330 | 0.719 |
| Fatigue score | 6.5 ± 1.8 | 6.8 ± 1.9 | 6.3 ± 2.0 | 0.450 | 0.639 | 6.1 ± 1.7 | 5.2 ± 1.6 | 6.1 ± 1.9 | 0.377 | 0.686 |
| Systolic blood pressure (mmHg) | 143.5 ± 7.2 | 142.8 ± 7.5 | 141.9 ± 7.0 | 0.323 | 0.725 | 140.5 ± 6.9 | 138.9 ± 7.1 | 141.2 ± 6.8 | 0.356 | 0.699 |
| Diastolic blood pressure (mmHg) | 88.5 ± 5.8 | 87.9 ± 5.6 | 87.3 ± 5.9 | 0.286 | 0.752 | 86.5 ± 5.5 | 85.2 ± 5.3 | 86.8 ± 5.7 | 0.383 | 0.682 |
| Body weight (kg) | 58.2 ± 6.8 | 58.8 ± 7.2 | 57.9 ± 7.0 | 0.110 | 0.896 | 57.5 ± 6.6 | 57.8 ± 7.0 | 57.8 ± 6.9 | 0.074 | 0.929 |
| BMI (kg/m²) | 23.9 ± 2.3 | 24.2 ± 2.5 | 23.8 ± 2.4 | 0.194 | 0.824 | 23.6 ± 2.2 | 23.7 ± 2.4 | 23.7 ± 2.4 | 0.125 | 0.883 |

**Note.** Data are presented as mean ± SD. F and P values refer to cross-sectional group comparisons across the three groups at the corresponding time point and do not represent the time × group interaction from the mixed-design ANOVA.

**Supplementary Table S7. Intention-to-treat sensitivity analysis using last observation carried forward**

| **Outcome** | **Per-protocol result** | **ITT-LOCF result** | **Interpretation** |
| --- | --- | --- | --- |
| Stroop incongruent accuracy | F(2,76) = 4.916, p = 0.010 | F(2,87) = 4.31, p = 0.016 | Remained significant |
| Flanker incongruent accuracy | F(2,76) = 4.683, p = 0.012 | F(2,87) = 4.05, p = 0.021 | Remained significant |
| Forward Digit Span | F(2,76) = 5.128, p = 0.008 | F(2,87) = 4.72, p = 0.011 | Remained significant |
| Backward Digit Span | F(2,76) = 6.409, p = 0.003 | F(2,87) = 5.57, p = 0.005 | Remained significant |
| POMS score | F(2,76) = 3.149, p = 0.049 | F(2,87) = 2.73, p = 0.071 | Attenuated to a marginal level |
| Fatigue score | F(2,76) = 8.212, p < 0.001 | F(2,87) = 6.91, p = 0.002 | Remained significant |
| Systolic blood pressure | F(2,76) = 2.547, p = 0.085 | F(2,87) = 2.02, p = 0.139 | Remained non-significant |
| Diastolic blood pressure | F(2,76) = 1.868, p = 0.161 | F(2,87) = 1.54, p = 0.221 | Remained non-significant |
| Body weight | F(2,76) = 0.207, p = 0.814 | F(2,87) = 0.18, p = 0.836 | Remained non-significant |
| BMI | F(2,76) = 0.337, p = 0.715 | F(2,87) = 0.29, p = 0.749 | Remained non-significant |

**Note.** ITT-LOCF = intention-to-treat analysis using the last observation carried forward approach. The per-protocol analysis included 79 completers, whereas the ITT-LOCF analysis included all 90 randomized participants. Baseline values were carried forward for participants with missing post-intervention data.

**Supplementary Table S8. Pre-to-post changes and between-group change differences with 95% confidence intervals**

| **Outcome** | **Baduanjin change 95% CI** | **Square dance change 95% CI** | **Control change 95% CI** | **Baduanjin vs Control 95% CI** | **Square dance vs Control 95% CI** | **Baduanjin vs Square dance 95% CI** |
| --- | --- | --- | --- | --- | --- | --- |
| Stroop incongruent accuracy, % | +2.5 [1.1, 3.9] | +1.3 [-0.2, 2.8] | +0.1 [-1.3, 1.5] | +2.4 [0.5, 4.3] | +1.2 [-0.8, 3.2] | +1.2 [-0.8, 3.2] |
| Flanker incongruent accuracy, % | +2.9 [1.1, 4.7] | +2.7 [0.8, 4.6] | +0.2 [-1.5, 1.9] | +2.7 [0.3, 5.1] | +2.5 [0.1, 4.9] | +0.2 [-2.3, 2.7] |
| Forward Digit Span | +0.52 [0.18, 0.86] | +0.33 [0.02, 0.64] | -0.07 [-0.36, 0.22] | +0.59 [0.15, 1.03] | +0.40 [-0.02, 0.82] | +0.19 [-0.25, 0.63] |
| Backward Digit Span | +0.49 [0.17, 0.81] | +0.28 [-0.04, 0.60] | -0.09 [-0.38, 0.20] | +0.58 [0.15, 1.01] | +0.37 [-0.06, 0.80] | +0.21 [-0.24, 0.66] |
| POMS score | -4.4 [-7.6, -1.2] | -4.9 [-8.4, -1.4] | -0.8 [-2.9, 1.3] | -3.6 [-7.2, 0.0] | -4.1 [-7.9, -0.3] | +0.5 [-4.3, 5.3] |
| Fatigue score | -0.4 [-0.7, -0.1] | -1.6 [-2.2, -1.1] | -0.2 [-0.7, 0.3] | -0.2 [-0.8, 0.4] | -1.4 [-2.1, -0.7] | +1.2 [0.5, 1.9] |

**Note.** CI = confidence interval. Change values were calculated as post-intervention minus baseline. Between-group change differences were calculated as the first named group minus the second named group. Positive values indicate greater increases or smaller decreases in the first named group, whereas negative values indicate greater decreases or smaller increases in the first named group.

**Supplementary Table S9. Effect sizes for time × group interactions**

| **Outcome** | **F value** | **P value** | **Partial η²** | **95% CI** |
| --- | --- | --- | --- | --- |
| Stroop incongruent accuracy | 4.916 | 0.010 | 0.115 | 0.015–0.241 |
| Flanker incongruent accuracy | 4.683 | 0.012 | 0.110 | 0.011–0.235 |
| Forward Digit Span | 5.128 | 0.008 | 0.119 | 0.019–0.248 |
| Backward Digit Span | 6.409 | 0.003 | 0.144 | 0.037–0.279 |
| POMS score | 3.149 | 0.049 | 0.076 | 0.000–0.194 |
| Fatigue score | 8.212 | <0.001 | 0.178 | 0.062–0.315 |

**Note.** Partial η² values and their 95% confidence intervals describe the magnitude and precision of time × group interaction effects. The outcomes shown are those with statistically significant time × group interactions in the per-protocol analysis.
